# Chemical Characterization and Antioxidant Properties of Ethanolic Extract and Its Fractions from Sweet Potato (*Ipomoea batatas* L.) Leaves

**DOI:** 10.3390/foods9010015

**Published:** 2019-12-23

**Authors:** Chengcheng Zhang, Daqun Liu, Liehong Wu, Jianming Zhang, Xiaoqiong Li, Weicheng Wu

**Affiliations:** 1 Food Science Institute, Zhejiang Academy of Agricultural Sciences, Hangzhou 310021, China; zccwsf@126.com (C.Z.); daqun.liu@hotmail.com (D.L.); zjm1990523@163.com (J.Z.); 0707lianlan@163.com (X.L.); 2 Institute of Crops and Nuclear Technology Utilization, Zhejiang Academy of Agricultural Sciences, Hangzhou 310021, China; wulh@zaas.ac.cn

**Keywords:** sweet potato leaves, antioxidant, phenolic compounds, fractions, caffeoylquinic acids

## Abstract

Sweet potato (*Ipomoea batatas* L.) leaf is a natural source of phenolic compounds with strong antioxidant activity and potential utility as an antioxidant. The aim of this study was to evaluate the polyphenol composition and antioxidant activities of ethanol extracts and their various solvent-partitioned fractions (petroleum ether, ethyl acetate, and aqueous fraction) from sweet potato leaves and petioles. Seven caffeoylquinic acid (CQA) derivatives and four flavonoids were detected in sweet potato leaves by HPLC-ESI-MS. The total phenolic content (TPC) and total flavonoid content (TFC) in leaf (112.98 ± 4.14 mg gallic acid equivalent (GAE)/g of dried extract, 56.87 ± 5.69 mg rutin equivalent (RE)/g of dried extract) was more than ten times higher than in petiole (9.22 ± 2.67 mg GAE/g of dried extract, 3.81 ± 0.52 mg RE/g of dried extract). The antioxidant contents of ethyl acetate fractions increased dramatically relative to those of crude extracts for both leaves and petioles. Purification using solvent partition with ethyl acetate increased TPC and TFC of crude extracts, especially the CQA derivatives including 3,4-dicaffeoylquinic acid, 3,5-dicaffeoylquinic acid, 4,5-dicaffeoylquinic acid, and 3,4,5-tricaffeoylquinic acid. Meanwhile, the ethyl acetate fractions with the highest CQA content were associated with the highest scavenging activities towards 2,2-diphenyl-1-picrylhydrazyl (DPPH) and higher ferric ion reducing antioxidant power (FRAP)-reducing power.

## 1. Introduction

Sweet potato (*Ipomoea batatas* L.) is a food crop from the morning glory family of Convolvulaceae. It is widely consumed around the world [1]. Apart from its roots, the young leaves and petiole (shoot) are often eaten as greens. “Zhecaishu 726” is a sweet potato cultivar with edible leaves and stems and is cultivated in the Zhejiang Academy of Agricultural Sciences. The sweet potato leaves are rich in polyphenols, proteins, vitamins, minerals, and other functional microcomponents [2,3]. Of these, phenolic compounds including caffeoylquinic acid (CQA) derivatives (mainly mono-CQA, di-CQA and 3,4,5-triCQA) [4], flavonoids (e.g., quercetin, myricetin, luteolin, and apigenin, etc.) [5,6], and anthocyanins [7] are the predominant biofunctional components. Sweet potato leaf polyphenols, especially di-CQA and 3,4,5-triCQA, have strong antioxidant capacities: Free radical scavenging, metal chelation, and inhibition of lipid peroxidation [8,9,10]. Liu et al. [11] studied the antioxidant activity of Jishu No. 18 sweet potato leaves cultivar and demonstrated that its ferric ion reducing antioxidant power (FRAP)-reducing power was almost 6.14, 3.37, and 9.43 times higher than that of the common vegetables like spinach, broccoli, and green cabbage, respectively [12]. In addition, the cellular and in vivo pharmacological evaluation of sweet potato leaf extract exhibited a wide range of health-promoting biological activities including antioxidative, anticancer, antibacterial, antidiabetic, and anti-inflammation [9,13,14,15]. Sweet potato leaves are thus nutritional and functional foods.

Currently, 95–98% of sweet potato leaves in China are discarded as waste with low value; the remaining 2–5% are mainly used for livestock [16], which leads to a huge waste of resources and creates environmental pollution problems. However, sweet potato leaves are excellent raw materials for the isolation of phenolic compounds, which demonstrate high antioxidant activity and can be incorporated into food products as nutritional supplements [17], food preservatives [18], and/or natural antioxidants [19]. In this context, recovery of these widely available and low-cost phenolic sources from sweet potato leaves could not only improve their added value, but also solve the ecological problem that these residues cause. It is thus essential to explore extraction processes to obtain maximum yields of these substances. Fu et al. [19] found that the type of extracting solvents greatly impacts the recovery and antioxidant activities of sweet potato leaf polyphenols: 50% (v/v) acetone and 70% ethanol are efficient solvents to recover polyphenols from sweet potato leaves. However, the crude polyphenol extracts of sweet potato leaves using single aqueous organic solvents always contain chlorophyll, proteins, polysaccharides, and other impurities that limit the application of sweet potato leaf polyphenols [20]. Fluid–fluid partition using solvents of different polarity is an efficient purification method for plant polyphenols [21]. However, phenolic compounds present in different plant material have their own unique polarities and chemical characteristics [22], so it is still not clear which solvent is more effective for purifying the polyphenols from a target plant. For example, Yang et al. [23] determined the bioactive phenolic components of extracts and purified fractions obtained from cultivated artichoke. The results showed that purified extract, the ethyl acetate fraction, revealed the highest total phenolic and total flavonoid contents, and also showed the strongest antioxidant activity compared to crude extract. Meanwhile, Wu et al. [24] compared the chemical composition of petroleum ether, ethyl acetate, *n*-butanol, and aqueous fractions from the ethanolic extract of *Lysimachia christinae*, and noted that *n*-butanol fractions had the highest total flavonoid (39.4 ± 4.55 mg rutin equivalent (RE)/g of extract), total phenolic (41.1 ± 3.07 mg gallic acid equivalent (GAE)/g of extract), and total polysaccharide (168.1 ± 7.07 mg glucose equivalent /g of extract). To the best of our knowledge, the purification and separation of polyphenol fractions from sweet potato leaf crude extracts and their chemical composition and antioxidant properties remain unclear. Therefore, the objective of the study was to determine the polyphenol composition and antioxidant activities of the ethanol extracts and their various solvent-partitioned fractions (petroleum ether, ethyl acetate, and aqueous fraction) from sweet potato leaves.

## 2. Materials and Methods

### 2.1. Chemicals and Reagents

Standards of rutin, gallic acid, hyperoside, kaempferol-3-*O*-glucoside, quercetin-3-*O*-hexoside, luteolin-7-*O*-glucoside, 3-caffeoylquinic acid (3-CQA), 3,4-dicaffeoylquinic acid (3,4-diCQA), 3,5-dicaffeoylquinic acid (3,5-diCQA), 4,5-dicaffeoylquinic acid (4,5-diCQA), and 3,4,5-tricaffeoylquinic acid were purchased from Yuanye Biotechnology Co., Ltd. (Shanghai, China). Folin–Ciocalteu reagent, 6-hydroxy-2,5,7,8-tetramethylchroman-2-carboxylic acid (Trolox), 2,2-diphenyl-1-picrylhydrazyl (DPPH), and 2,4,6-tris (2-pyridyl)-*s*-triazine (TPTZ) were purchased from Sigma–Aldrich (Shanghai, China). HPLC-grade methanol was purchased from Merck (Darmstadt, Germany). All other analytical-grade chemicals and reagents used in the study: Ethanol, petroleum ether, ethyl acetate, acetone, ether, chloroform, *n*-butanol, methanol, formic acid, phenol, sulfuric acid, sodium nitrite (NaNO_2_), aluminum nitrate (Al(NO_3_)_3_·9H_2_O), sodium hydroxide (NaOH), sodium carbonate, 3,5-dinitrosalicylic acid (DNS), and ferric chloride were purchased from Sinopharm Chemical Reagent Co., Ltd. (Shanghai, China).

### 2.2. Extraction and Fractionation

**Figure 1:**
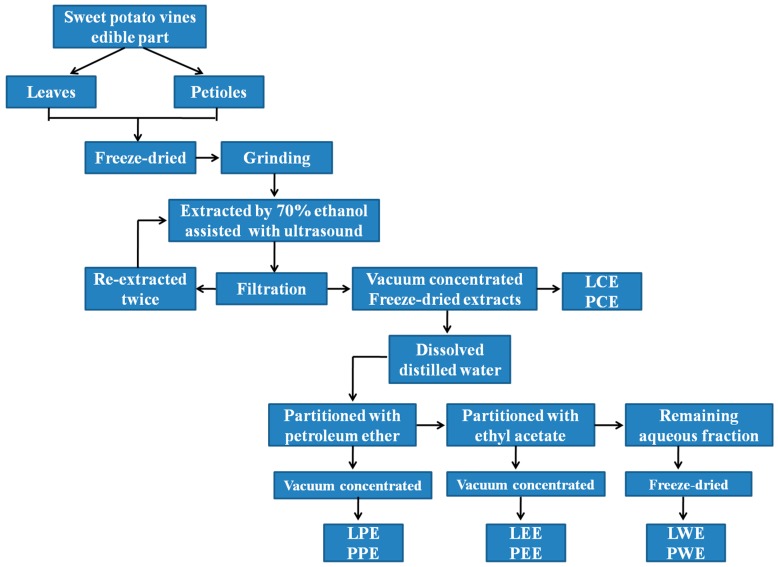
Flow diagram showing the fractionation of ethanolic extract in sweet potato leaves. LCE, PCE, LPE, PPE, LEE, PEE, LWE, and PWE mean leaf crude extract, petiole crude extract, leaf petroleum ether extract, petiole petroleum ether extract, leaf ethyl acetate extract, petiole ethyl acetate extract, leaf water extract, and petiole water extract, respectively.

Fresh sweet potato vines (Zhecaishu 726) were collected from the farmland of Zhejiang Academy of Agricultural Sciences (Hangzhou, China) in August 2019, and identified by Professor Wu from the Institute of Crops and Nuclear Technology Utilization, Zhejiang Academy of Agricultural Sciences. The sweet potato vines were separated into leaves and petioles after nonedible stems were removed. Fresh leaves and petioles were lyophilized (SCIENTZ-18N; Ningbo Scientz Biotechnology Co., Ltd., Ningbo, China) and ground to a fine powder through an 80-mesh sieve. Then, 20 g of dried leaves and petiole powder were separately extracted with 70% ethanol (400 mL) by ultrasound-assisted extraction for approximately 30 min at 50 °C, and the supernatant was filtered. Afterward, the residue was re-extracted twice with 70% ethanol as described above. The supernatants were collected, concentrated in a rotary evaporator (RE-52; Yarong Instruments Co., Ltd., Shanghai, China), and freeze-dried (SCIENTZ-18N; Ningbo Scientz Biotechnology Co., Ltd., Ningbo, China) to obtain a crude ethanol extract designated as leaf crude extract (LCE) and petiole crude extract (PCE). The LCE and PCE were separately dissolved in 200 mL distilled water and then partitioned with different solvents. Briefly, partition with petroleum ether yielded the petroleum ether fraction, designated as leaf petroleum ether extract (LPE) and petiole petroleum ether extract (PPE). Ethyl acetate yielded the ethyl acetate fraction, designated as leaf ethyl acetate extract (LEE) and petiole ethyl acetate extract (PEE). The final aqueous fraction was designated as leaf water extract (LWE) and petiole water extract (PWE). After evaporation and lyophilization, the petroleum ether fraction, ethyl acetate fraction, and remaining water fraction were weighed and stored at −20 °C until analysis. The fractionation of the ethanolic extract is detailed in Figure 1.

The extraction yield of extracts or fractions from sweet potato leaves was calculated according to the formula: Extraction yield = (mg dried extract matter)/(g dried leaf or petiole). The extract was dissolved in 80% methanol to make a solution of 1.0 mg/mL for total flavonoid content, total phenolic content, antioxidant activity, and chromatographic analysis.

### 2.3. Identification and Quantification of Polyphenol Compounds

#### 2.3.1. Identification of Polyphenol Compounds by HPLC-ESI-MS

Identification of polyphenol compounds in sweet potato leaves was performed using an Agilent 1200 liquid chromatography (LC) system equipped with a degasser, a binary pump, a thermostatted HiP-ALS auto-sampler, a diode array detector (DAD), and electrospray ionization (ESI) source (Agilent Technologies, Santa Clara, CA, USA). Chromatographic separation used a ZORBAX SB-C18 column (4.6 × 250 mm, 5 μm) (Agilent Technologies, Santa Clara, CA, USA) kept at 35 °C. The flow rate was 1 mL/min, and all samples were filtered through a 0.22 μm syringe filter prior to injection (10 μL). The 0.1% formic acid in deionized water and methanol were defined as solvents A and B, respectively. A gradient was generated under the following conditions: 0–6 min, 10–25% B; 6–15 min, 25–35% B; 15–23 min, 35–40% B; 23–30 min, 40–80% B; 30–34 min, 80–100% B; 34–38 min, 100–10%; and 38–45 min, 10% B. The detection wavelengths were set at 280 nm, 320 nm, and 360 nm. Electrospray ionization mass spectrometry (ESI-MS) detection used a negative ion mode with mass acquisition between 200 and 1500 Da. Nitrogen was used as the desolvation gas at a flow rate of 800 L/h and desolvation temperature at 300 °C. The identification of phenolic compounds was performed through comparison of their retention times and the MS data with those of pure compounds or reported data.

#### 2.3.2. Quantitative Analysis of Polyphenol Compounds by HPLC

Quantitative analysis of polyphenol compounds used a ZORBAX SB-C18 column (4.6 × 250 mm, 5 μm) (Agilent Technologies, Santa Clara, CA, USA) in a Waters 2695 HPLC system equipped with 2998 UV/Visible detector (Waters Corporation, Milford, MA, USA). The elution conditions used for quantitative analysis were the same as those used for qualitative analysis, and the peaks were detected at 320 nm and 360 nm. Quantification of the polyphenol compounds was done using external standard methods.

### 2.4. Determination of Total Flavonoid Content

The total flavonoid content (TFC) was assayed as described by Jia et al. [25] with minor modifications using rutin as a standard. Here, 1 mL of the extracted samples were put in a 10 mL volumetric flask followed by 0.4 mL 5% (*w*/*v*) NaNO_2_ solutions. After 6 min, 0.4 mL 10% (*w*/*v*) Al (NO_3_)_3_ was added to the flask for another 6 min reaction. Finally, 4 mL 5% (*w*/*v*) NaOH solution was added to the mixture and kept for 15 min before the absorbance at 510 nm was measured on a spectrophotometer (UV-2600, Unico Instruments Co., Ltd., Shanghai, China). The results were expressed as mg rutin equivalent/g dried extract (mg RE/g dried extract).

### 2.5. Determination of Total Phenolic Content

Total phenolic content (TPC) was determined according to the Folin–Ciocalteu method with minor modifications [26]. Briefly, 0.4 mL of each extract was transferred to a 10 mL volumetric flask containing 2.4 mL distilled water. Then, 0.4 mL 10% (*v*/*v*) Folin–Ciocalteu reagent and 1.2 mL 7.5% (*w*/*v*) sodium carbonate was added to each flask. The absorbance at 765 nm was measured by a spectrophotometer after 2 h of standing at room temperature. All results were expressed as mg gallic acid equivalent/g dried extract (mg GAE/g dried extract).

### 2.6. Determination of Sugar Content

#### 2.6.1. Total Polysaccharide Content

The 1.0 g of each extract was accurately weighed and dissolved in distilled water to obtain 100 mg/mL sample solution. Four times the volume of 95% ethanol was mixed with the sample solution to precipitate crude polysaccharide (4 °C, 12 h), and the precipitates were centrifuged at 5000× *g* for 15 min and then sequentially washed with anhydrous ethanol, acetone, and ether. Subsequently, the crude polysaccharide was deproteinized by the Sevage method (chloroform: *n*-butanol = 4:1) [27]. Finally, a polysaccharide was obtained by freeze-drying, and the phenol-sulfuric acid method was used to determine the contents of total polysaccharides [28]. The results were expressed as mg glucose equivalent/g dried extract (mg GE/g dried extract).

#### 2.6.2. Reducing Sugar Content

The extract was dissolved in distilled water to make a sample solution of 1.0 mg/mL. The reducing sugar content was assayed by the DNS method with the same analytical setup, and using glucose as a standard [29]. Reducing sugar content was expressed as mg glucose equivalent/g dried extract (mg GE/g dried extract).

### 2.7. Antioxidant Capacity Measurement

#### 2.7.1. DPPH Assay

The DPPH free radical scavenging activity was carried out according to Chen et al. [30] with some modifications. A series of concentrations of the extract sample at 5, 10, 25, 50, 125, and 500 mg/mL was prepared. Briefly, 1 mL of each extract was allowed to react with 2 mL of 0.1 mmol/L DPPH solution for 30 min in the dark before the absorbance was read at 517 nm. The radical scavenging activity was calculated as % Inhibition = [(AB − AA)/AB] × 100, where AA was the absorption of tested extract solution and AB was the absorption of blank sample.

#### 2.7.2. FRAP Assay

The FRAP assay was evaluated according to previous studies with minor modifications [30]. The FRAP reagent was freshly prepared by mixing 300 mM acetate buffer (pH 3.6) and 10 mM TPTZ solution prepared in 40 mmol/L HCl and 20 mM ferric chloride at a ratio of 10:1:1 (*v*/*v*/*v*). The 0.5 mL of extracts and 4.5 mL of FRAP reagent were transferred into a vial and incubated at 37 °C for 30 min. The absorbance was read at 593 nm and the results were expressed as mg Trolox equivalent/g dried extract (mg TE/g dried extract).

### 2.8. Statistical Analysis

All experiments were run at least in triplicate. The results were expressed as mean ± standard deviation (SD) for each group. Statistical analyses were performed using SAS 9.1 software (SAS Institute, Cary, NC, USA). All data were analyzed using the ANOVA procedure with significance at *p* < 0.05 level. Correlations among data obtained were found using standard Pearson’s correlation with SPSS 20.0 software (SPSS Inc., Chicago, IL, USA). The principal component analysis (PCA) was performed with Canoco 4.5 software (Biometris, Plant Research International, Wageningen, The Netherlands).

## 3. Results and Discussion

### 3.1. Extraction Yield of Fractions

The extraction yield of sweet potato leaf crude extracts and their fractions are shown in Table 1. The extraction yield of dry matter in the extract of petioles (PCE, 501.4 mg/g) was greater than in leaves (LCE, 344.1 mg/g). The extraction yields of fractions purified by solvent partition from PCE and LCE showed significant differences. Water fractions showed the highest extraction yields (219.8 and 470.5 mg/g in LWE and PWE, respectively), followed by petroleum ether fraction (111.2 and 23.9 mg/g in LPE and PPE, respectively) and ethyl acetate fractions (12.5 and 6.3 mg/g in LEE and PEE, respectively). These results suggest that sweet potato leaves may possess a number of water-soluble components such as proteins, minerals, and carbohydrates [2].

### 3.2. Characterization and Quantification of Phenolic Compounds

**Figure 2:**
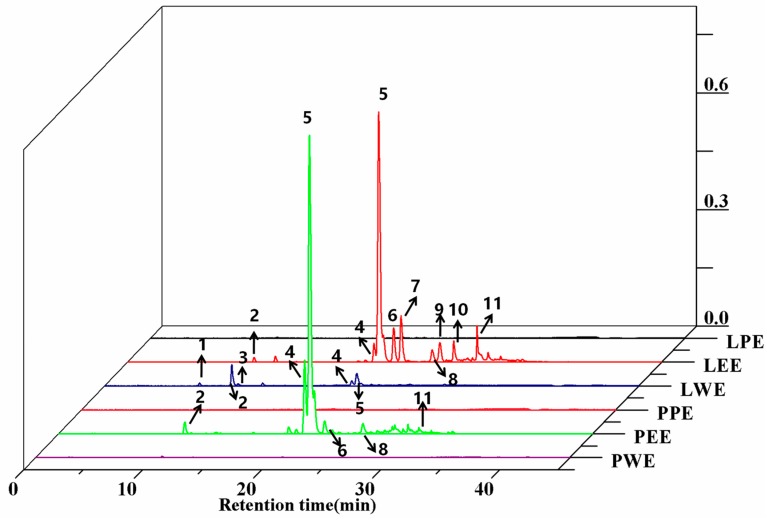
HPLC chromatograms of phenolic compounds from the crude extract and their fractions in sweet potato leaves. LPE, PPE, LEE, PEE, LWE, and PWE mean leaf petroleum ether extract, petiole petroleum ether extract, leaf ethyl acetate extract, petiole ethyl acetate extract, leaf water extract, and petiole water extract, respectively.

The identification and peak assignment of the polyphenol constituents in ethanol extracts and different solvent fractions used retention times and mass spectral data with the corresponding authentic standards. The molecular formula, retention time, and mass spectral data of the identified compounds are summarized and described in Table 2. A total of 11 polyphenols were detected in sweet potato leaves, including seven CQA derivatives and four flavonoids. The identification of most of those CQAs has been reported in previous studies [19]. Peaks 1, 2, and 3 have similar parent ion peaks at *m*/*z* 352.9 [M-H]^−^. These were assigned as mono-CQAs with the same formula C_16_H_18_O_9_ and identified as 5-caffeoylquinic acid, 3-caffeoylquinic acid, and 4-caffeoylquinic acid, respectively. Three types of di-CQAs with a deprotonated [M-H]^−^ at *m*/*z* 514.9 and a typical fragment ion at *m*/*z* 353 were identified to be 3,4-dicaffeoylquinic acid (peak 4), 3,5-dicaffeoylquinic acid (peak 5) and 4,5-dicaffeoylquinic acid (peak 8), respectively. Similarly, peak 11 ([M-H]^−^ at *m*/*z* 676.9) was positively defined as 3,4,5-tricaffeoylquinic acid by comparing with the corresponding standard. Peaks 6 and 7 were identified as quercetin derivatives based on their deprotonated molecular ions at *m*/*z* 463 and an aglycone fragment ion at *m*/*z* 301. This *m*/*z* 301 peak was due to the loss of galactoside and hexoside moieties. The similar pseudomolecular ion peak at *m*/*z* 447.0 [M-H]^−^ of peaks 9 and 10 were identified as luteolin-7-*O*-glucoside and kaempferol-3-*O*-glucoside. These two compounds were reported for the first time in sweet potato leaves. Figure 2 and Table 2 show that all 11 compounds were detected in LCE; six compounds were found in PCE, excluding compounds 1, 3, 7, 9, and 10. This indicates that the phenolic profiles in petioles significantly varied across leaf samples.

Table 3 shows the quantification of the identified compounds in the crude extract and their fractions from sweet potato leaves. The leaf extract had more phenolic compounds than the petiole portion. Table 3 shows that the total CQA derivative levels in LCE and PCE were 56.43 and 5.76 mg/g dried extract, respectively. Total flavonoids levels were 6.56 and 0.29 mg/g dried extract, respectively. Jang et al. [31] reported that the levels of CQA derivatives in sweet potato leaves were more than ten times higher than those in the petioles. Therefore, the sweet potato leaf portion is a promising potential source of phenolic compounds. Table 3 shows that the compounds were further distributed after sequential partitioning with petroleum ether and ethyl acetate due to their corresponding solubility and solvent polarity. For sweet potato leaf samples, most of the detected mono-CQAs with relative higher polarity were enriched in the water fractions, and the ethyl acetate fractions had the most di-CQAs, 3,4,5-tricaffeoylquinic acid, and flavonoids. The petroleum fractions had the lowest number and content of phenolic compounds. Most of the detected phenolic compounds in petioles, including 3-CQA, 3,4-diCQA, 3,5-diCQA, 4,5-diCQA, 3,4,5-triCQA, and hyperoside, were enriched in the ethyl acetate fractions (Table 3). The extraction efficiency of a compound is strongly influenced by its solubility and the polarity of the extracting solvent [23]. Here, solvent partition using ethyl acetate allows the phenolic compounds to increase dramatically. For example, 3,5-dicaffeoylquinic acid was the predominant polyphenol in sweet potato leaves with the highest content in the LEE (229.10 ± 32.59 mg/g dried extract) and PEE (263.64 ± 2.67 mg/g dried extract). It was also 12.3 and 72.2 times that in the LCE and PCE, indicating that ethyl acetate is an efficient solvent for enriching polyphenols from sweet potato leaves.

### 3.3. TPC, TFC, Total Polysaccharides, and Reducing Sugar Contents

The total flavonoid, total phenolic, total polysaccharide, and reducing sugar contents of each fraction from sweet potato extract are shown in Table 4. The levels of reducing sugar in sweet potato leaf crude extract (52.93 ± 1.70 mg GE/g) were significantly lower than those in petiole (403.78 ± 7.09 mg GE/g). However, the TPC, TFC, and polysaccharide content in LCE was 112.98 ± 4.14 mg GAE/g, 56.87 ± 5.69 mg RE/g, and 49.41 ± 3.86 mg GE/g, respectively. This is much higher than that in PCE with a value of 9.22 ± 2.67 mg GAE/g, 3.81 ± 0.52 mg RE/g, and 11.14 ± 0.94 mg GE/g, respectively. The results were consistent with the quantitative results showing that sweet potato leaves provide a richer source of phenolic compounds than petioles. Fu et al. [19] reported similar results where the TPC and TFC of sweet potato were 135.9 mg GAE/g and 5.8 mg RE/g, respectively, using ethanol as the extraction solvent.

The fractions purified by solvent partition from LCE and PCE exhibited significant differences in phenolic, flavonoid, and sugar contents. Ethyl acetate fractions LEE and PEE displayed the highest values of TPC with 338.34 ± 21.81 and 375.44 ± 9.78 mg GAE/g, respectively. The TFC was 110.14 ± 4.10 and 127.12 ± 2.53 mg RE/g, respectively. The highest total amounts of total polysaccharide and reducing sugar contents were observed in the water fractions. Polyphenols and flavonoids had lower polarities and could be compatible with medium polarity solvents like ethyl acetate [23]. These results were consistent with a previous report suggesting that the ethyl acetate fractions had the highest TPC and TFC, and that the water fractions exhibited the lowest amounts [32].

### 3.4. DPPH Radical Scavenging and FRAP-Reducing Activity

**Figure 3:**
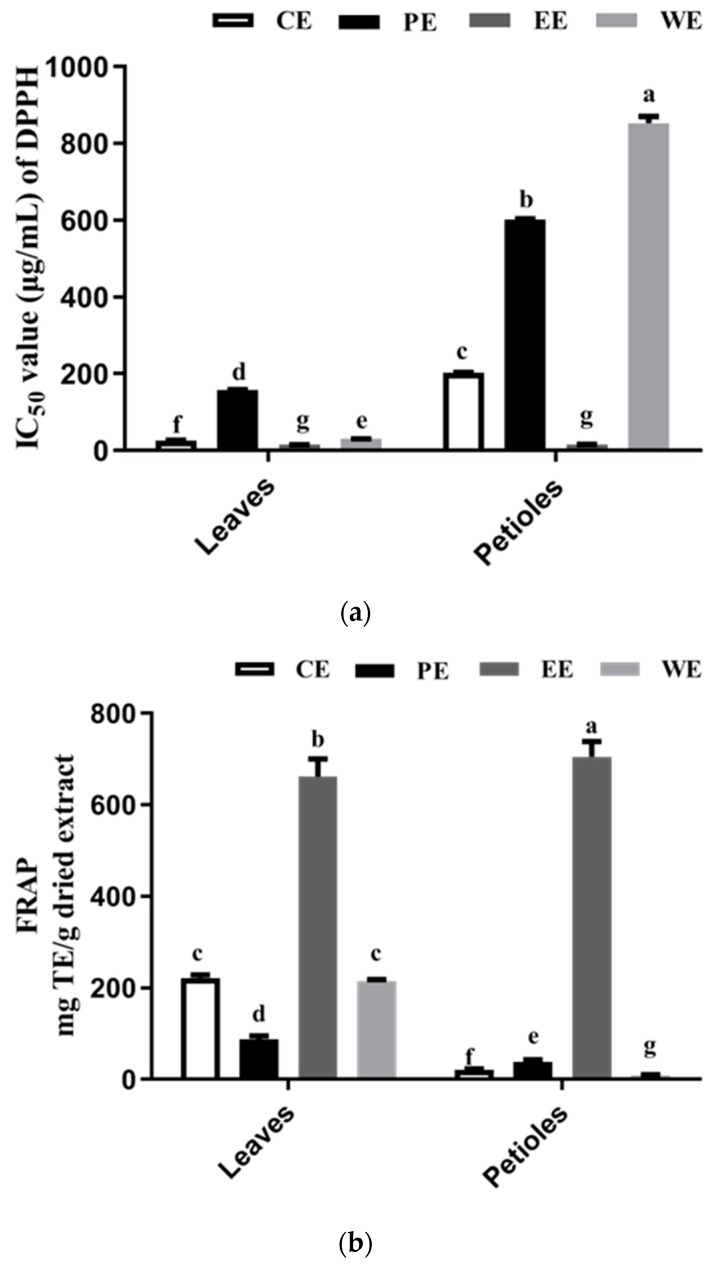
Antioxidant activity of the crude extract and their fractions from sweet potato leaves using two kinds of assays: (**a**) IC_50_ values of 2,2-diphenyl-1-picrylhydrazyl (DPPH) free radical scavenging activity for leaves and petioles extract; (**b**) Ferric ion reducing antioxidant power (FRAP)-reducing power. For each column, values followed by the same letter are not significantly different (*p* < 0.05). CE, PE, EE, and WE mean crude extract, petroleum ether extract, ethyl acetate extract, and water extract, respectively.

The results of DPPH radical scavenging activities of sweet potato leaf extracts and fractions are shown as IC_50_ in Figure 3a. LCE (IC_50_, 26.76 μg/mL) exhibited a distinctly higher DPPH radical scavenging activity than PCE (IC_50_, 202.02 μg/mL). In a previous study, Jang et al. [31] reported a similar overall trend of DPPH results. The EC_50_ values of DPPH for petioles were about ten-fold those in the leaves. As shown, the DPPH radical scavenging activity of both ethyl acetate fractions (LEE and PEE) are expected to increase relative to those of the crude extracts (LCE and PCE) after liquid/liquid extraction. The ethyl acetate fractions (LEE and PEE) exhibited the strongest DPPH scavenging activity (Figure 3a), and the petroleum ether fractions (PPE and LPE) showed the weakest DPPH scavenging effect. Our results were consistent with previous studies suggesting that an increase of the DPPH free radical scavenging activity in the plant extract suggests more phenolic compounds (TFC and TPC) [33,34,35].

Figure 3b shows that the FRAP-reducing power of samples is PEE > LEE > LCE > LWE > LPE > PPE > PCE > PWE. This order is consistent with the DPPH scavenging activity. Here, ethyl acetate fractions PEE and LEE showed the highest FRAP-reducing power with 705.03 ± 10.69 and 661.61 ± 12.76 mg TE/g dried extract, respectively. These values may be associated with a high level of TPC, especially with CQA derivatives (including di-CQA and 3,4,5-triCQA) as the major antioxidant in the ethyl acetate extract. These antioxidant compounds can reduce the oxidized intermediates by donating electrons; thus, they have antioxidant activities [36]. These results indicate that the higher reducing power of PEE and LEE may be related to both the higher phenolic content and the stronger electron-donating abilities of the individual phenolic compounds.

### 3.5. Statistical Analysis

**Figure 4:**
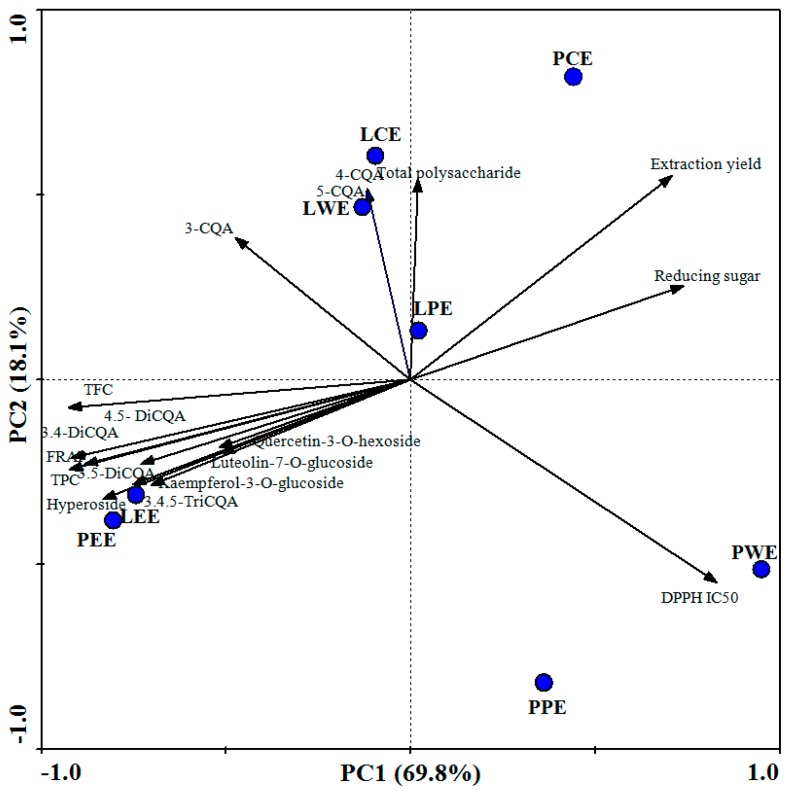
Biplot of first and second component from principal component analysis. LCE, PCE, LPE, PPE, LEE, PEE, LWE, and PWE mean leaf crude extract, petiole crude extract, leaf petroleum ether extract, petiole petroleum ether extract, leaf ethyl acetate extract, petiole ethyl acetate extract, leaf water extract, and petiole water extract.

The biplot obtained from the first two principal components (PCs) collectively explained 87.9% of the total variance in the data set (Figure 4). Each point corresponded to an extract sample in the score plot. Measured parameters (TPC, TFC, DPPH, FRAP, CQA derivatives, flavonoids, total polysaccharide, reducing sugar, and extraction yield) were displayed by vectors. Here, LEE and PEE were found on the lower left quadrant. They were characterized by high TPC, TFC, di-CQA, 3,4,5-triCQA, flavonoids, and FRAP-reducing power. The LWE and LCE in the upper left quadrant represents the mono-CQAs including 5-caffeoylquinic acid, 3-caffeoylquinic acid, and 4-caffeoylquinic acid. The PCE was characterized by a high extraction yield. It also had a high content of total polysaccharide and reducing sugar, while the PPE and PWE were mostly characterized by the high IC_50_ values of DPPH.

In PCA, angles between vectors lower than 90° indicate positive associations, and angles near 180° indicate negative associations between variables [23]. Variables such as TPC, TPC, CQA derivatives, and flavonoids formed angles lower than 90° with FRAP. Those formed near 180° with DPPH (IC_50_) indicate a positive association with antioxidant capacity values. Phenolic compounds can contribute considerably to the overall antioxidant capacity [37]. The values of TPC and TFC of different extracts were highly correlated with FRAP (*r* = 0.997 and 0.951). The relationship between individual phenolic compounds in sweet potato leaves and antioxidant activity was also analyzed. The 4,5-diCQA showed the highest correlation coefficient (*r* = 0.992), followed by 3,5-diCQA (*r* = 0.973), 3,4-diCQA (*r* = 0.838), and 3,4,5-triCQA (*r* = 0.824). The antioxidant activity of sweet potato leaves could be mainly attributed to the high contents of di-CQA and 3, 4, 5-triCQA: These have more hydroxyl groups in their molecular structure, making them more active than mono-CQA [10]. In addition, flavonoids in sweet potato leaves showed weaker antioxidant activities (*r* = 0.860, 0.586, 0.597, and 0.571 for hyperoside, quercetin-3-*O*-hexoside, luteolin-7-*O*-glucoside, and kaempferol-3-*O*-glucoside, respectively). The glycosylation of a flavonoid reduces its antioxidant capacity because this process decreases the number of hydroxyl groups [38]. Thus, we speculate that the special high content of di-CQA and 3,4,5-triCQA in ethyl acetate fractions underlies the better antioxidant ability.

## 4. Conclusions

This study analyzed the polyphenol composition and antioxidant activities of various fractions obtained from sweet potato leaves and petioles. The ethyl acetate fractions displayed the highest phenolic and flavonoid contents with the best antioxidant activity. Through correlation analysis of phytochemical contents and antioxidant capacities, the representative antioxidant components in sweet potato leaves were identified as phenolic compounds, especially caffeoylquinic acid (CQA) derivatives including 4,5-diCQA, 3,5-diCQA, 3,4-diCQA, and 3,4,5-triCQA. In addition, sweet potato leaf presented higher phenolic compounds as compared to the petiole. These results indicate that sweet potato leaves are excellent sources for polyphenols and functional food ingredients. Future research should focus on the purification of the bioactive polyphenols in sweet potato leaf and carry out in-depth studies for their health-promoting activities.

## Figures and Tables

**Table 1:** Extraction yield of sweet potato leaves’ crude extracts and their fractions.

Samples	Extraction Yield (mg/g)	Samples	Extraction Yield (mg/g)
LCE	344.1	PCE	501.4
Fraction—LPE	111.2	Fraction—PPE	23.9
Fraction—LEE	12.5	Fraction—PEE	6.3
Fraction—LWE	219.8	Fraction—PWE	470.5

LCE, PCE, LPE, PPE, LEE, PEE, LWE and PWE mean leaf crude extract, petiole crude extract, leaf petroleum ether extract, petiole petroleum ether extract, leaf ethyl acetate extract, petiole ethyl acetate extract, leaf water extract, and petiole water extract, respectively.

**Table 2:** Chemical composition of the crude extract and their fractions in sweet potato leaves using HPLC-MS in negative mode.

Peak No.	Rt (min)	[M-H]^−^ (*m*/*z*)	MS Fragments (*m*/*z*)	Molecular Formula	Compound	Samples
1	8.54	352.94	227, 707	C_16_H_18_O_9_	5-Caffeoylquinic acid	LCE, LWE
2	11.49	352.84	275, 707	C_16_H_18_O_9_	3-Caffeoylquinic acid	LCE, LEE, LWE, PCE, PEE
3	12.13	352.92	227, 707	C_16_H_18_O_9_	4-Caffeoylquinic acid	LCE, LWE
4	22.67	514.93	353, 227	C_25_H_24_O_12_	3,4-Dicaffeoylquinic acid	LCE, LEE, LWE, PCE, PEE
5	23.21	514.86	353, 227	C_25_H_24_O_12_	3,5-Dicaffeoylquinic acid	LCE, LEE, LWE, PCE, PEE
6	24.78	463.02	300, 301, 363	C_21_H_20_O_12_	Hyperoside	LCE, LPE, LEE, PCE, PEE
7	25.43	462.97	300, 301, 315	C_21_H_20_O_12_	Quercetin-3-*O*-hexoside	LCE, LPE, LEE
8	27.57	514.90	607, 353	C_25_H_24_O_12_	4,5-Dicaffeoylquinic acid	LCE, LEE, LWE, PCE, PEE
9	28.01	447.01	227, 284, 363	C_21_H_20_O_11_	Luteolin-7-*O*-glucoside	LCE, LEE
10	28.60	447.03	249, 284, 353	C_21_H_20_O_11_	Kaempferol-3-*O*-glucoside	LCE, LEE
11	29.87	676.94	515, 365	C_34_H_30_O15	3,4,5-Tricaffeoylquinic acid	LCE, LEE, PCE, PEE

LCE, PCE, LPE, PPE, LEE, PEE, LWE, and PWE mean leaf crude extract, petiole crude extract, leaf petroleum ether extract, petiole petroleum ether extract, leaf ethyl acetate extract, petiole ethyl acetate extract, leaf water extract, and petiole water extract, respectively.

**Table 3:** Quantitative results of the identified compounds in the crude extract and their fractions from sweet potato leaves (mg/g dried extract).

Peak	Compound	LCE	LPE	LEE	LWE	PCE	PPE	PEE	PWE
1	5-Caffeoylquinic acid	2.82 ± 0.21 ^b^	ND	ND	4.89 ± 0.85 ^a^	ND	ND	ND	ND
2	3-Caffeoylquinic acid	16.77 ± 0.89 ^b^	ND	6.18 ± 0.77 ^d^	27.35 ± 2.66 ^a^	0.21 ± 0.05 ^e^	ND	12.95 ± 0.13 ^c^	ND
3	4-Caffeoylquinic acid	2.29 ± 0.32 ^b^	ND	ND	4.21 ± 0.59 ^a^	ND	ND	ND	ND
4	3,4-Dicaffeoylquinic acid	11.64 ± 0.45 ^c^	ND	22.89 ± 3.20 ^b^	8.70 ± 0.68 ^d^	1.16 ± 0.16 ^e^	ND	83.28 ± 1.66 ^a^	ND
5	3,5-Dicaffeoylquinic acid	18.69 ± 1.34 ^c^	ND	229.10 ± 32.59 ^b^	17.24 ± 1.47 ^c^	3.65 ± 0.69 ^d^	ND	263.64 ± 2.67 ^a^	ND
6	Hyperoside	2.46 ± 0.22 ^d^	3.22 ± 0.13 ^c^	33.58 ± 0.58 ^a^	ND	0.29 ± 0.06 ^e^	ND	15.16 ± 1.39 ^b^	ND
7	Quercetin-3-*O*-hexoside	2.71 ± 0.44 ^b^	3.36 ± 0.17 ^b^	44.43 ± 0.88 ^a^	ND	ND	ND	ND	ND
8	4,5-Dicaffeoylquinic acid	3.40 ± 0.77 ^b^	ND	16.51 ± 1.82 ^a^	4.74 ± 0.58 ^b^	0.19 ± 0.03 ^c^	ND	15.73 ± 0.64 ^a^	ND
9	Luteolin-7-*O*-glucoside	0.75 ± 0.17 ^b^	ND	21.77 ± 0.55 ^a^	ND	ND	ND	ND	ND
10	Kaempferol-3-*O*-glucoside	0.64 ± 0.10 ^b^	ND	18.46 ± 0.48 ^a^	ND	ND	ND	ND	ND
11	3,4,5-Tricaffeoylquinic acid	0.82 ± 0.04 ^c^	ND	21.62 ± 1.63 ^a^	ND	0.11 ± 0.02 ^d^	ND	7.75 ± 0.12 ^b^	ND

Results are expressed as mean ± standard deviation of three replicates. For each line, means followed by different letters are significantly different (*p* < 0.05). LCE, PCE, LPE, PPE, LEE, PEE, LWE, and PWE mean leaf crude extract, petiole crude extract, leaf petroleum ether extract, petiole petroleum ether extract, leaf ethyl acetate extract, petiole ethyl acetate extract, leaf water extract, and petiole water extract, respectively. ND: Not detected.

**Table 4:** Total phenolic, total flavonoid, total polysaccharide, and reducing sugar content of the crude extract and their fractions from sweet potato leaves.

	Samples	Total Flavonoid Content (mg RE/g of Dried Extract)	Total Phenolic Content (mg GAE/g of Dried Extract)	Total Polysaccharide Content (mg GE/g of Dried Extract)	Reducing Sugar Content (mg GE/g of Dried Extract)
Leaves	LCE	56.87 ± 5.69 ^d^	112.98 ± 4.14 ^d^	49.41 ± 3.86 ^b^	52.93 ± 1.70 ^d^
	LPE	16.56 ± 1.39 ^e^	52.63 ± 2.24 ^e^	ND	ND
	LEE	110.14 ± 4.10 ^b^	338.34 ± 21.81 ^b^	ND	ND
	LWE	80.53 ± 2.05 ^c^	125.61 ± 4.01 ^c^	89.90 ± 5.85 ^a^	91.39 ± 2.82
Petioles	PCE	3.81 ± 0.52 ^g^	9.22 ± 2.67 ^f^	11.14 ± 0.94 ^d^	403.78 ± 7.09 ^b^
	PPE	11.75 ± 2.46 ^f^	47.14 ± 4.38 ^e^	ND	ND
	PEE	127.12 ± 2.53 ^a^	375.44 ± 9.78 ^a^	ND	ND
	PWE	1.52 ± 0.23 ^h^	2.90 ± 0.18 ^g^	15.01 ± 1.62 ^c^	417.37 ± 10.36 ^a^

Results are expressed as mean ± standard deviation of three replicates. For each column, means followed by different letters are significantly different (*p* < 0.05). LCE, PCE, LPE, PPE, LEE, PEE, LWE, and PWE mean leaf crude extract, petiole crude extract, leaf petroleum ether extract, petiole petroleum ether extract, leaf ethyl acetate extract, petiole ethyl acetate extract, leaf water extract, and petiole water extract. ND: Not detected.
